# Diagnostic Value of Epigastric Pains

**Published:** 1851-03

**Authors:** 


*Diagnostic Value of Epigastric Pains.*—According to Wunderlich, ('Handbuch der Pathologie und Therapie,') the following points are worthy of attention, in reference to pains in the epigastric region:

Gastric pains in persons whose digestion and appetite are unaffected, and which are not exacerbated by hunger or food, most probably depend upon the spinal cord, or on some organ adjacent to the stomach. Pains which are diminished by strong pressure may be set down to neuralgia. Pains which are distinctly increased when the hand is placed gently on the stomach, but which are not proportionally aggravated by firm or abrupt pressure, are either imaginary or sympathetic. Pains continue for days or weeks, or recurring at definite periods without any obvious cause; also pains which come on suddenly with great severity and disappear as rapidly, are, probably, due to gastric neuralgia; they may depend on gaseous distention. Pains arising in the scrobiculus cordis and radiating in various directions, may arise from cardialgia, rheumatism of the abdominal wall, or peritonitis. Gastric pains, which are suspended by food, depend on neuralgic affection, or the presence of parasites. Pains which exist, both while ordinary food is taken and when fasting, but which disappear when stimulating food or drink is taken, depend on anemia of the stomach. Pains which are increased by the smallest quantity of food, indicate probable gastritis or degeneration. If pains supervene an hour or two after meal-time, we fear disease of the pylorus. [This assertion will needlessly alarm a large proportion of dyspeptics.—Ed.] Lancinatings are sometimes remarked in cancer.—*Ib.*

